# Application of antibody-drug conjugates in locally advanced or metastatic urothelial carcinoma: mechanisms, treatment-related adverse events, and management strategies

**DOI:** 10.3389/fphar.2026.1771653

**Published:** 2026-03-31

**Authors:** Yunduo Fan, Guipeng Wang, Shang Xu, Huaixi Ge, Yuanxiao Li, Xinning Wang

**Affiliations:** 1 Department of Urology, The Affiliated Hospital of Qingdao University, Qingdao, China; 2 Department of Nephrology, The Affiliated Hospital of Qingdao University, Qingdao, China

**Keywords:** antibody-drug conjugates, immune checkpoint inhibitors, immunotherapy, treatment-related adverse events, urothelial carcinoma

## Abstract

Locally advanced or metastatic urothelial carcinoma (la/mUC) is a highly aggressive malignancy with a poor prognosis. For decades, platinum-based chemotherapy has remained the cornerstone of first-line treatment. In recent years, antibody-drug conjugates (ADCs), leveraging their ability to precisely target tumor sites, have emerged as important therapeutic options, advancing into second-line and even first-line settings. The advent of ADCs has not only provided new alternatives for patients ineligible for chemotherapy but also offered a variety of treatment regimens. These agents enhance therapeutic efficacy while being adaptable to different patient subtypes, achieving satisfactory outcomes with relatively manageable safety profiles. Consequently, ADCs have rapidly become a clinical star and a research hotspot. Enfortumab Vedotin (EV), Sacituzumab Govitecan (SG), and Disitamab Vedotin (DV) are currently among the most studied ADCs in la/mUC. However, as the clinical experience with ADCs is still relatively nascent, accumulating knowledge regarding their safety is ongoing. It is crucial for clinicians to understand the mechanisms of action of ADCs and to master the management, including prevention and mitigation strategies, of treatment-related adverse events (TRAEs) associated with different ADC agents.

## 1 Introduction

Urothelial carcinoma (UC), also known as transitional cell carcinoma, most commonly occurs in the bladder, followed by the renal pelvis, ureter, and urethra (Puente et al., 2025). Bladder cancer, the most prevalent form of UC, is not only the most common malignant tumor of the urinary system but also ranks among the top 10 most common malignancies globally, and is the fourth most common cancer in males (Lopez-Beltran et al., 2024). UC is highly aggressive and carries a poor prognosis, presenting a significant therapeutic challenge when it progresses to locally advanced or metastatic urothelial carcinoma (la/mUC) (Siegel et al., 2024; Roviello et al., 2024). Although platinum-based chemotherapy remains the current first-line standard, its relatively severe treatment-related adverse events (TRAEs) and restrictive eligibility criteria limit its overall efficacy in the treatment of la/mUC (Moussa et al., 2024). In recent years, targeted therapies, notably antibody-drug conjugates (ADCs), have broadened the treatment landscape and expanded therapeutic options for patients with la/mUC (Compérat et al., 2022). Currently, the FDA has approved the combination of Enfortumab Vedotin (EV) and pembrolizumab as a first-line treatment for all la/mUC patients (Powles et al., 2024a; Powles et al., 2024b).

**FIGURE 1:**
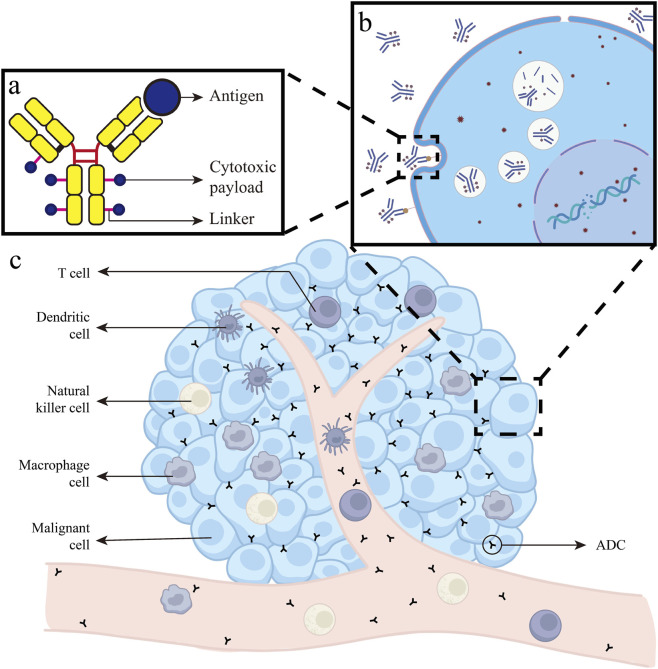
**(a)** Schematic diagram of ADC structure. The ADC is primarily composed of an antibody that recognizes a specific target antigen, a cytotoxic payload, and a chemical linker connecting the two components. **(b)** Mechanism of ADC action *in vivo*. After administration, the ADC circulates systemically, specifically binds to its target antigen on the cancer cell surface, and is subsequently internalized. Within the cell, the ADC is trafficked to lysosomes, where degradation leads to the release of the cytotoxic payload. This released agent ultimately induces cancer cell death by causing DNA strand breaks and disrupting microtubule assembly. **(c)** Role of ADC within the Tumor Microenvironment. ADCs can modulate the tumor microenvironment by promoting the maturation and activation of antigen-presenting cells and enhancing the infiltration and aggregation of effector immune cells.

In 2000, the FDA first approved the ADC gemtuzumab ozogamicin for adult acute myeloid leukemia, marking the beginning of the era of ADC-based targeted cancer therapy. Subsequently, ADCs have gradually been applied to solid tumors. ADCs are referred to as “biological missiles” consisting of an antibody, a chemical linker, and a cytotoxic payload. They achieve targeted therapeutic effects by binding to specific target antigens on the surface of cancer cells (Fu et al., 2022). A schematic diagram of the ADC structure and its mechanism of targeting tumor cells is shown in Figure 1. Through the monoclonal antibody’s specific binding to a particular antigen, ADCs achieve “precision guidance,” which locates cancer cells while significantly reducing off-target toxic effects. After the monoclonal antibody binds to the target cell, the ADC is internalized, forming early endosomes that subsequently develop and ultimately fuse with lysosomes. Within the lysosomes, the cytotoxic payload is released, primarily causing apoptosis or death of the target cell by damaging DNA or interfering with tubulin formation (Kim and Chang, 2022). Simultaneously, ADCs can diffuse through the cell membrane to neighboring cells, exerting cytotoxic effects regardless of whether these cells express the target antigen. This “bystander effect” enables ADCs to extend their therapeutic impact within the tumor microenvironment (Jin et al., 2022). Furthermore, combination therapy with immune checkpoint inhibitors (ICIs) has been a major research focus for ADCs in recent years (Xu et al., 2023; Chen et al., 2023). The cytotoxic payload carried by ADCs can directly influence the maturation and activation of dendritic cells and enhance the infiltration of CD8^+^ T cells within tumors, thereby improving anti-tumor immune responses and potentiating the efficacy of ICIs.

However, factors such as low-level expression of the target antigen, low antibody affinity, premature cleavage of the chemical linker, the nature of the cytotoxic payload, and the drug’s half-life may all contribute to the occurrence of TRAEs, posing challenges to the clinical application of ADCs (Donaghy, 2016). This review primarily summarizes the TRAEs associated with ADCs targeting la/mUC in clinical use and discusses relevant management strategies, aiming to provide assistance to clinicians.

## 2 The common three in ADCs for la/mUC

According to current research, the primary targets of ADCs for la/mUC are categorized into three major groups: Nectin-4, Trop-2, and Her-2. The corresponding therapeutic agents are EV, Sacituzumab Govitecan (SG), and Disitamab Vedotin (DV) (Zeng et al., 2025), all of which have completed phase I-III clinical trials and have accumulated substantial clinical evidence, as detailed in Supplementary Table S1.

### 2.1 Enfortumab Vedotin

**FIGURE 2:**
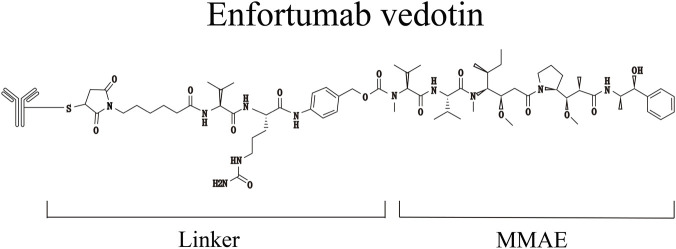
The molecular structural formula of EV.

EV (Figure 2) consists of a monoclonal antibody targeting Nectin-4 conjugated to the cytotoxic payload monomethyl auristatin E (MMAE). Nectin-4 is a transmembrane cell adhesion molecule involved in intercellular adhesion; in tumor cells, it also participates in processes such as invasion, migration, proliferation, angiogenesis, epithelial-mesenchymal transition, and DNA repair (Samanta and Almo, 2014). Studies indicate that Nectin-4 is abundantly expressed in embryos and the placenta, maintained at low levels in normal adults, and shows limited expression in normal tissues such as the skin, bladder, breast, esophagus, stomach, and salivary glands. In contrast, it is overexpressed in various solid tumors, including lung, breast, ovarian, pancreatic, and bladder cancers, particularly in urothelial carcinoma, where overexpression is observed in up to 60% of cases (Challita-Eid et al., 2016). A multicenter study confirmed that Nectin-4 amplification can serve as a genetic predictive biomarker for sensitivity to EV and long-term survival in patients with metastatic bladder cancer (Klümper et al., 2024). MMAE belongs to the auristatin family and induces apoptosis by binding to and disrupting microtubules.

EV-101 was a phase I dose-escalation/expansion study. With respect to dose concentration, it was observed that as the dose concentration increased, the incidence and severity of TRAEs correspondingly rose. At a dose of 1.0 mg/kg, 11% of patients required dose reductions due to TRAEs; at the recommended phase II dose of 1.25 mg/kg, this proportion reached 35%. At the recommended dose, the most common TRAEs included fatigue, alopecia, decreased appetite, dysgeusia, nausea, peripheral sensory neuropathy, pruritus, and diarrhea. Among these patients, 34% experienced grade ≥3 TRAEs (Rosenberg et al., 2020).

In the subsequent EV-103 study, patients were enrolled to receive EV in combination with pembrolizumab (Hoimes et al., 2023), and later to compare EV plus pembrolizumab versus EV monotherapy (O’Donnell et al., 2023). Among the combination therapy groups, the most common TRAEs were peripheral sensory neuropathy, fatigue, alopecia, and maculopapular rash. The incidence of grade ≥3 TRAEs was similar between the two cohorts, both exceeding 60%. Notably, consistent with the findings from EV-101, peripheral sensory neuropathy was the most frequent TRAE leading to dose reduction or treatment discontinuation. The incidence of grade ≥3 TRAEs observed in both cohorts of this study was higher than that previously reported for EV monotherapy. Based on prior research, we consider that hypothyroidism and pneumonitis observed in the combination groups were more likely attributable to pembrolizumab-related TRAEs (Leroy et al., 2017; Wang et al., 2019).

EV-201, a global phase II clinical study, primarily investigated the efficacy and safety of sequential treatment with EV in patients with la/mUC who had previously received platinum-based chemotherapy and ICI therapy (Rosenberg et al., 2019) or ICI therapy alone (Yu et al., 2021). The most common TRAEs across both cohorts were fatigue, alopecia, decreased appetite, and peripheral sensory neuropathy. The incidence of grade ≥3 TRAEs was similar between the two cohorts (54% vs. 55%), with neutropenia being the most frequent grade ≥3 TRAE in both. Peripheral sensory neuropathy also remained the most common TRAE leading to dose reduction or treatment discontinuation. The study results indicated that prior sequential therapies did not impact the subsequent safety profile of EV.

EV-301 further compared the efficacy and safety of EV monotherapy with chemotherapy (Powles et al., 2021). The study demonstrated that EV monotherapy was significantly superior to chemotherapy in terms of efficacy. Regarding safety, there was no significant difference between the two groups in the incidence of TRAEs (93.9% vs. 91.8%) or in the incidence of grade ≥3 TRAEs (52.4% vs. 50.5%). Notably, the profiles of TRAEs differed between the two groups. In the chemotherapy group, grade ≥3 TRAEs primarily manifested as bone marrow suppression, including neutropenia, leukopenia, and anemia. In contrast, in the EV group, maculopapular rash, fatigue, and peripheral sensory neuropathy were more commonly observed (Rosenberg et al., 2023).

EV-302 demonstrated that EV combined with pembrolizumab had superior efficacy and safety to platinum-based chemotherapy. The combination group’s common TRAEs were peripheral sensory neuropathy, pruritus, and alopecia; grade ≥3 TRAEs (55.9%) were primarily maculopapular rash, hyperglycemia, and neutropenia. Notably, although both groups exhibited bone marrow suppression manifestations, represented by neutropenia, previous studies indicate that bone marrow suppression associated with EV is typically observed in combination therapy settings (Rosenberg et al., 2019; Yu et al., 2021), whereas chemotherapy is more frequently associated with bone marrow suppression and cytopenias across all three hematopoietic lineages (Visweshwar et al., 2018; Kasi and Grothey, 2018). Additionally, in the combination group, peripheral sensory neuropathy remained the most common TRAE leading to discontinuation of EV (Powles et al., 2024b).

### 2.2 Sacituzumab Govitecan

**FIGURE 3:**
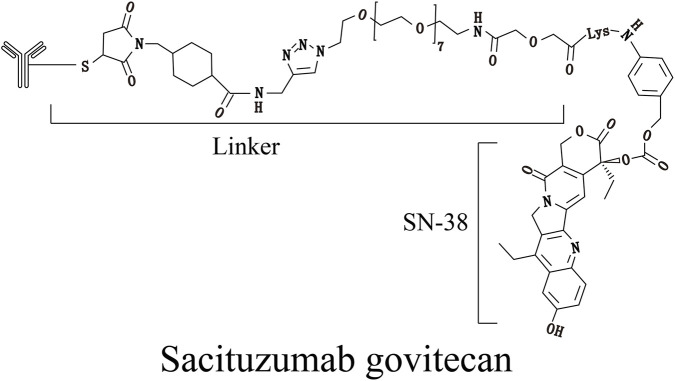
The molecular structural formula of SG.

SG (Figure 3) is composed of a monoclonal antibody targeting Trop-2 conjugated to the payload SN-38. Recent studies have shown that Trop-2 acts as a significant tumor-promoting factor by modulating calcium ion signaling pathways, cyclin expression, and reducing fibronectin adhesion, thereby promoting tumor cell growth, proliferation, and metastasis (Goldenberg et al., 2018). Similar to Nectin-4, Trop-2 is widely expressed in many epithelial tumors, such as squamous cell carcinoma, urothelial carcinoma, breast cancer, prostate cancer, pancreatic cancer, and ovarian cancer. Notably, high expression of Trop-2 is associated with advanced stages and lymph node metastasis in colorectal cancer, gastric adenocarcinoma, and papillary thyroid carcinoma; conversely, low Trop-2 expression correlates with advanced urothelial carcinoma, high-grade breast cancer, and high-grade papillary renal cell carcinoma (Dum et al., 2022). The payload SN-38 in SG is an active metabolite of the topoisomerase I inhibitor irinotecan. It induces double-strand DNA breaks during the S phase of the cell cycle, thereby inhibiting tumor cell proliferation and dissemination (Goldenberg and Sharkey, 2019). Unlike EV, which is currently specifically applied to urothelial carcinoma, SG is currently widely tested in breast cancer, lung cancer, gastric cancer, urothelial carcinoma, and other malignancies (Cardillo et al., 2015; Bardia et al., 2017; Faltas et al., 2016).

First, the IMMU-132-01 study investigated the use of SG in patients with various solid tumors, including 45 patients (9%) with mUC (Bardia et al., 2021). Across the solid tumor population, the most commonly observed TRAEs were nausea, diarrhea, neutropenia, fatigue, and alopecia. Grade ≥3 TRAEs occurred in 59.6% of patients, including febrile neutropenia, diarrhea, vomiting, neutropenia, and nausea. Notably, neutropenia was the TRAE most frequently leading to treatment discontinuation or interruption, which distinguishes it from the profile of EV but bears resemblance to that of conventional chemotherapy.

TROPHY-U-01, a phase II trial, evaluated SG as monotherapy or combined with pembrolizumab in la/mUC. In the two cohorts of the SG monotherapy study (Petrylak et al., 2024; Tagawa et al., 2021; Loriot et al., 2024), the most common TRAEs were similar, primarily including diarrhea, nausea, and fatigue. The common grade ≥3 TRAEs in both cohorts encompassed neutropenia, leukopenia, and anemia, consistent with previous findings. In the combination cohort, common TRAEs included diarrhea, nausea, neutropenia, anemia, asthenia, alopecia, and fatigue, with 61% of patients experiencing grade ≥3 TRAEs, among which neutropenia remained the most frequent (Grivas et al., 2024). Nearly half of the patients (46%) in the SG monotherapy cohorts developed treatment-related neutropenia of any grade, while febrile neutropenia was relatively less common (10%). Neutropenia also represented the most common reason for treatment interruption, discontinuation, or dose reduction.

TROPiCS-04 is a phase III clinical trial comparing the efficacy and safety of SG versus treatment of physician’s choice (TPC), which consisted of various chemotherapy regimens, in patients with la/mUC (Powles et al., 2025). A comparative analysis showed that the incidence of grade ≥3 TRAEs was higher in the SG group than in the TPC group (67% vs. 35%). Regarding the neutropenia of particular interest, it remained not only the most common grade ≥3 TRAE in the SG group (35%) but also the leading cause of treatment-related mortality (64%). The higher incidences of grade ≥3 neutropenia, grade ≥3 infections secondary to neutropenia, and treatment-related deaths may be associated with the relatively low utilization rate of granulocyte colony-stimulating factor (G-CSF) support in the primary prophylaxis setting.

### 2.3 Disitamab Vedotin

**FIGURE 4:**
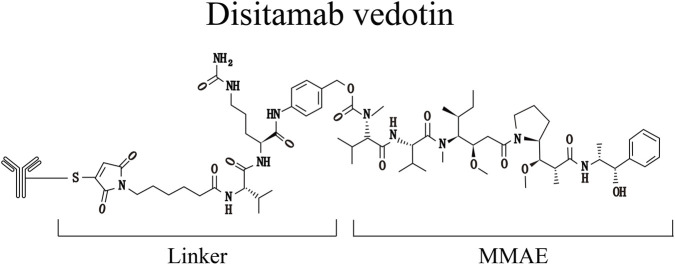
The molecular structural formula of DV.

DV (Figure 4), also known as RC48-ADC, consists of a monoclonal antibody targeting Her-2 conjugated to the same payload as EV, namely MMAE. Her-2 is a tyrosine kinase receptor that promotes growth and participates in cell proliferation, peritumoral angiogenesis, and tumorigenesis. It has been confirmed to be widely overexpressed in solid tumors such as breast cancer, gastric cancer, ovarian cancer, and endometrial cancer, and is strongly associated with poor prognosis and increased tumor aggressiveness (Hudis, 2007; Jones et al., 2024). Previous studies have indicated that Her-2 can serve as a prognostic biomarker for non-muscle invasive bladder cancer (NMIBC). Moreover, Her-2 is highly expressed in NMIBC tumor tissues and is correlated with advanced pathological features, disease recurrence, and unfavorable prognosis (Zhao et al., 2015; Moustakas et al., 2020).

First, regarding DV monotherapy in patients with la/mUC, Sheng et al. conducted sequential studies, enrolling two cohorts of patients to investigate the efficacy and safety of DV (Sheng et al., 2024; Sheng et al., 2021). The findings from these two studies revealed closely aligned safety profiles. The most common TRAEs included peripheral sensory neuropathy, hypoesthesia, alopecia, leukopenia, neutropenia, and fatigue. Notably, both studies reported only up to grade 3 TRAEs (58.0% & 54.2%), with no grade 4 or 5 TRAEs observed. Furthermore, the most common grade 3 TRAE in both cohorts was hypoesthesia/peripheral sensory neuropathy, which also represented the most frequent reason for treatment discontinuation. Additionally, increased aspartate aminotransferase (AST) and alanine aminotransferase (ALT) levels were observed in 42.1% and 35.5% of patients, respectively, suggesting potential hepatotoxicity associated with DV. However, dedicated studies on this aspect are currently lacking.

In studies investigating DV combined with ICIs in patients with la/mUC, the safety profiles observed in most studies appear more manageable compared to those reported for EV, SG, and even DV monotherapy. Notably, some studies reported no grade ≥3 TRAEs or TRAEs leading to treatment discontinuation/dose reduction (Ng et al., 2025; Wei et al., 2023; Zhu et al., 2024; Yao et al., 2025; Zhou et al., 2025; Zhang T. et al., 2025). Common TRAEs associated with DV plus ICIs therapy include rash & pruritus, asthenia, peripheral sensory neuropathy, alopecia, leukopenia, neutropenia, and gastrointestinal symptoms (nausea and anorexia). It is also noteworthy that indicators of potential hepatotoxicity, such as increased ALT, AST, and gamma-glutamyl transferase (GGT), have been observed. Grade ≥3 TRAEs reported include gastrointestinal bleeding complicated by intestinal obstruction, rash, pneumonia, increased GGT, and peripheral sensory neuropathy. One comparative study found a substantial disparity in the incidence of grade ≥3 TRAEs (0% in the combination group vs. 73.08% in the chemotherapy group) (Zhang T. et al., 2025).

In studies investigating DV monotherapy or DV combined with ICIs, we observed a safety profile similar to that of DV plus ICIs. Specifically, the incidence of grade ≥3 TRAEs was substantially lower compared to related studies of EV and SG, and the rate of dose modifications due to TRAEs was also reduced (Xu et al., 2023; Chen et al., 2023; Wang et al., 2024; Ge et al., 2025; Chen et al., 2024; Wang et al., 2025). Common TRAEs included anemia, nausea, asthenia, peripheral sensory neuropathy, leukopenia, and anorexia, along with reported indicators of hepatotoxicity. Interestingly, while anticancer therapies—including targeted therapies, immunotherapies, and chemotherapies—typically pose challenges in patients with poor renal function due to potential negative impacts on the kidneys (Shahinian et al., 2017), Chen et al. found no increase in TRAEs among patients with unfavorable conditions or impaired renal function (Chen et al., 2024). Comparative results suggest that combination therapy carries a higher safety risk than DV monotherapy. For example, the incidence of grade ≥3 TRAEs was 27.8% in the combination group versus 11.1% in the monotherapy group in one study (Chen et al., 2023), and 14.8% versus 0% in another (Wang et al., 2025). Based on the evidence from DV/DV plus ICIs studies, it appears that while combination therapy offers efficacy advantages, it also introduces increased safety risks.

## 3 TRAEs based on different ADCs: incidence, characteristics, and related mechanisms

This section will focus on TRAEs as an entry point to systematically summarize the common and representative TRAEs associated with the three aforementioned ADCs, and attempt to provide a comprehensive overview of their incidence, characteristics, and potential underlying mechanisms—TRAEs that researchers often regard as requiring priority assessment and special attention. The incidences of key TRAEs for different ADCs, as discussed in the relevant sections above, are presented in Supplementary Table S2.

### 3.1 Rash associated with EV

In EV monotherapy, the incidence of rash ranges from approximately 15%–55%, with grade ≥3 rash occurring in approximately 1%–20% of cases, and the proportion of grade ≥3 events accounting for approximately 5%–50% of all rash events. When combined with Pembrolizumab, these figures are approximately 30%–50%, 10%–20%, and 20%–40%, respectively. Overall, the incidence of rash and the proportion of severe cases are relatively low, with rashes being more common in combination therapy with Pembrolizumab. Most rash events are mild to moderate in severity, and even severe cases can be alleviated or improved with appropriate management. The most common types of rash include maculopapular rash, erythematous rash, and eczema. Studies have documented that the onset of rash often occurs during the first treatment cycle, with a median time to onset of 0.7 months (IQR: 0.3–4.1 months). Two other datasets reported median onset times of 0.5 months (IQR: 0.3–0.9 months) and 0.43 months (IQR: 0.03–12.68 months), respectively. The median time to resolution was 1.0 months (IQR: 0.4–2.2 months) (Powles et al., 2024b; Rosenberg et al., 2020; Hoimes et al., 2023; O’Donnell et al., 2023; Powles et al., 2021; Rosenberg et al., 2023; Takahashi et al., 2020).

Given the moderate expression of Nectin-4 in keratinocytes and skin appendages of normal human skin, many studies consider rash, alopecia, pruritus, and dry skin as expected on-target toxicities. EV delivers MMAE to normal tissues via targeting of Nectin-4 expressed in these tissues, which is regarded as the mechanism underlying skin reactions, particularly rash (Hirotsu et al., 2021; Gazzoni et al., 2025). Meanwhile, Nectin-4 plays a role in adherens junctions and epithelial cohesion; its blockade and internalization may impair cell-cell adhesion and compromise skin barrier function, thereby also contributing to dermatitis-like reactions (Ebnet, 2008; Mühlebach et al., 2011).

### 3.2 Peripheral sensory neuropathy associated with EV & DV

Peripheral sensory neuropathy is a common TRAE associated with EV and DV. The underlying mechanism is attributed to their shared cytotoxic payload, MMAE (Fu et al., 2023).

The incidence of peripheral sensory neuropathy with EV monotherapy ranges from approximately 0%–50%, with grade ≥3 events occurring in approximately 0%–5% of cases, and the proportion of grade ≥3 events accounting for approximately 0%–15% of all cases. When combined with Pembrolizumab, these figures are approximately 50%, 1%–5%, and 1%–10%, indicating that while the overall incidence and severity are relatively low, peripheral neuropathy is more common in the combination setting (Powles et al., 2024b; Rosenberg et al., 2020; Hoimes et al., 2023; O’Donnell et al., 2023; Powles et al., 2021; Rosenberg et al., 2023; Takahashi et al., 2020). For DV, the incidence of peripheral sensory neuropathy is similar between monotherapy and combination therapy, with an overall range of approximately 10%–70% (mostly around 50%), grade ≥3 events in about 0%–20% of cases, and the proportion of grade ≥3 events accounting for 0%–30% of all cases. Unlike EV, where combination therapy elevates incidence, no significant differences are observed between DV monotherapy and combination regimens (Chen et al., 2023; Sheng et al., 2024; Sheng et al., 2021; Ng et al., 2025; Wei et al., 2023; Zhu et al., 2024; Yao et al., 2025; Zhou et al., 2025; Zhang T. et al., 2025; Wang et al., 2025). For both EV and DV, most events are sensory in nature, mild to moderate in severity, and generally low-grade and manageable. In most patients, symptoms had resolved or improved by the last follow-up.

For EV, studies have shown that the presence of pre-existing peripheral sensory neuropathy does not affect its incidence following EV treatment, whereas previous research has indicated that advanced age, underlying diseases, and other spinal cord or nerve-related conditions are associated with a higher risk of developing peripheral sensory neuropathy (Pace et al., 2021). Unlike rash, peripheral neuropathy typically begins after the second cycle, with a median time to onset of 2.4 months (IQR: 1.9–4.6 months). Two other datasets reported median onset times of 2.4 months (IQR: 1.2–3.6 months) and 2.81 months (IQR: 0.03–13.04 months), respectively. The median time to resolution was 5.2 months (IQR: 3.5–8.6 months).

Peripheral sensory neuropathy is generally considered to be a toxicity associated with MMAE. Researchers propose that MMAE accumulates in neural tissues either through bystander effects extending beyond the tumor or via non-specific uptake of the ADC, leading to peripheral neurotoxicity by impairing rapid anterograde axonal transport (Frachet et al., 2023). This represents a shared TRAE among ADCs with MMAE as the payload.

### 3.3 Hyperglycemia associated with EV

The incidence of hyperglycemia with EV monotherapy ranges from approximately 0%–10%, with grade ≥3 events occurring in approximately 0%–10% of cases; however, the proportion of grade ≥3 events accounts for over 50% of all hyperglycemia cases. When combined with Pembrolizumab, these figures are approximately 10%, 5%–10%, and over 50%, respectively. Overall, although the incidence of hyperglycemia is relatively low, the proportion of grade ≥3 events is as high as 50% or more. The incidence of hyperglycemia shows minimal difference between combination therapy and monotherapy, with previous systematic reviews and meta-analyses reporting similar findings (Cheng et al., 2025). Studies suggest that hyperglycemia occurs more frequently in patients with a body mass index ≥30 kg/m^2^ or in those with baseline hyperglycemia or diabetes mellitus. Furthermore, although most cases of hyperglycemia achieve significant resolution during subsequent treatment and follow-up, the resulting ketoacidosis is often a contributing factor to multiple organ failure and mortality in affected patients. Hyperglycemia typically occurs within the first cycle of treatment, with a median time to onset of 0.5 months (IQR: 0.5–0.5 months). Two other datasets reported median onset times of 0.62 months (IQR: 0.26–13.37 months) and 0.5 months (IQR: 0.5–1.0 months), respectively. The median time to resolution was 1.6 months (IQR: 0.7–1.6 months) (Powles et al., 2024b; Rosenberg et al., 2020; Hoimes et al., 2023; O’Donnell et al., 2023; Powles et al., 2021; Rosenberg et al., 2023; Takahashi et al., 2020).

The pathophysiology of EV-induced hyperglycemia is currently not fully understood, although it can resolve spontaneously (Hussain et al., 2025). Previous studies have suggested that Pembrolizumab may lead to elevated blood glucose through autoimmune-mediated destruction of pancreatic islet cells, a process resembling type 1 diabetes mellitus; however, this occurrence is relatively rare (Kotwal et al., 2019).

### 3.4 Neutropenia/febrile neutropenia associated with SG

The incidences of neutropenia and febrile neutropenia are generally similar between SG monotherapy and its combination with Pembrolizumab. The incidence of neutropenia is approximately 50%, and that of febrile neutropenia is approximately 10%. The incidence of grade ≥3 events is approximately 30%–50% for neutropenia and 5%–10% for febrile neutropenia, with the proportion of grade ≥3 events accounting for approximately 70% and 90%–100% of all cases, respectively. Thus, although the overall incidence is relatively low, the incidence and proportion of grade ≥3 events are notably high, particularly for febrile neutropenia, where the proportion of grade ≥3 events approaches or reaches 100%. The median time to first onset of neutropenia is 19 days (mean: 32.8 days), with a median duration of 8.5 days (mean: 12.4 days). For grade ≥3 neutropenia, the median time to first onset is 17 days (mean: 40.9 days), with a median duration of 8 days (mean: 8.4 days) (Bardia et al., 2021; Petrylak et al., 2024; Tagawa et al., 2021; Loriot et al., 2024; Grivas et al., 2024; Powles et al., 2025). Additionally, relevant studies have found that patients homozygous for UGT1A1 allele variants have an increased risk of TRAEs such as neutropenia and diarrhea. However, current evidence remains limited, and routine pre-screening for UGT1A1 genotype is not currently recommended (Dello et al., 2026).

Neutropenia increases the risk of infection and treatment-related complications. We note that the TROPiCS-04 study reported the occurrence of fatal events related to neutropenia. Among the 25 deaths in patients receiving SG monotherapy, 16 occurred in the setting of neutropenia, with 14 of these occurring within the first month of treatment. The study found that affected patients had a higher burden of risk factors associated with medical complications. Additional risk factors included age ≥65 years, extensive visceral disease, and renal impairment. The mechanism by which SG induces neutropenia is described in the following section (Powles et al., 2025).

### 3.5 Diarrhea associated with SG

The incidence of diarrhea with SG monotherapy ranges from approximately 50%–70%, with grade ≥3 events occurring in approximately 5%–15% of cases, and the proportion of grade ≥3 events accounting for 10%–30% of all diarrhea cases. When combined with Pembrolizumab, these figures are approximately 70%, 20%, and 30%, respectively. It can be observed that the severity of diarrhea is relatively lower compared to neutropenia. Furthermore, studies have found that, unlike neutropenia, diarrhea rarely leads to SG discontinuation and has a better prognosis with more favorable recovery outcomes, which may be attributed to the systemic rather than local release of the SN-38 metabolite. The median time to first onset of diarrhea is 14 days (mean: 29.5 days), with a median duration of 8 days (mean: 28.2 days). For grade ≥3 diarrhea, the median time to first onset is 15 days (mean: 67.3 days), with a median duration of 6 days (mean: 7.6 days) (Bardia et al., 2021; Petrylak et al., 2024; Tagawa et al., 2021; Loriot et al., 2024; Grivas et al., 2024; Powles et al., 2025).

Neutropenia and diarrhea induced by SG are primarily attributed to elevated systemic exposure to SN-38, the active metabolite of the parent compound irinotecan (de Man et al., 2018). UGT1A1 is the most critical enzyme responsible for inactivating SN-38. Similar to peripheral sensory neuropathy caused by MMAE, when SN-38 is released systemically due to instability in a small proportion of the ADC or bystander effects, genetic variants in UGT1A1 may result in reduced or absent enzymatic activity, thereby contributing to the occurrence of these TRAEs (Hulsh et al., 2022; Xu et al., 2026). Studies have also indicated that early-onset diarrhea occurring within 24 h post-administration is caused by irinotecan-mediated inhibition of acetylcholinesterase, which increases cholinergic activity, thereby impairing absorptive capacity and stimulating contraction of intestinal smooth muscle. Early-onset diarrhea can often be prevented or alleviated by administering anticholinergic agents (Xu et al., 2024).

### 3.6 Hepatotoxicity associated with DV

The reporting of DV-related hepatotoxicity varies across studies in terms of terminology and indicators. Overall, the incidence of liver function abnormalities with DV monotherapy ranges from approximately 10%–40%, with grade ≥3 events occurring in approximately 0%–5% of cases (a substantial proportion of which are reported as 0%), and the proportion of grade ≥3 events accounting for 0%–30% of all cases, with the higher end of this range observed when gamma-glutamyl transferase (GGT) is used as the reference indicator. When combined with Pembrolizumab, these figures are notably higher, ranging from 10% to 70%, 0%–15%, and 0%–20%, respectively. Overall, the incidence of DV-related hepatotoxicity is relatively low compared to other TRAEs of interest; however, in contrast to the minimal liver function impact observed with EV and SG, DV-related hepatotoxicity has garnered attention from researchers (Dong et al., 2025). Hepatotoxicity observed in DV-related studies also showed resolution or improvement during subsequent follow-up, demonstrating a manageable safety profile for DV (Chen et al., 2023; Sheng et al., 2024; Sheng et al., 2021; Ng et al., 2025; Wei et al., 2023; Zhu et al., 2024; Yao et al., 2025; Zhou et al., 2025; Zhang T. et al., 2025; Wang et al., 2025).

Regarding the mechanism of DV-induced liver function impairment, it has not yet been fully elucidated by current research. Based on the available studies, researchers have summarized potential mechanisms by which ADCs may cause hepatotoxicity, including but not limited to on-target toxicity, off-target toxicity, instability of linker and conjugation, receptor-mediated internalization, non-specific endocytosis, bystander effect, and immunogenicity-related hepatotoxicity (Dong et al., 2025).

## 4 Management strategies for common TRAEs: current landscape and future directions

There are numerous strategies for mitigating drug side effects, with common approaches including refinement at the drug design level, empirical preventive measures combined with active symptomatic management in clinical practice and emerging methods for TRAE risk stratification and prediction. The following section will elaborate on these three aspects.

### 4.1 Strategic improvement of the ADC framework

First, at the drug design level, premature cleavage of the linker, leading to the release of free payload, often results in the off-target attack of normal cells by ADCs, particularly rapidly dividing cells (Donaghy, 2016). Similarly, the bystander effect of ADCs can inadvertently damage normal tissues adjacent to tumor sites, contributing to the occurrence of TRAEs (Tolcher et al., 2003). Furthermore, the specific payload incorporated influences the toxicity profile of the ADC. For instance, ADCs with MMAE as the payload typically exhibit a higher incidence of peripheral neuropathy (Best et al., 2021), while those carrying SN-38 are more frequently associated with neutropenia (Satoh et al., 2013). Therefore, carefully selecting the payload to modulate its inherent toxicity can help reduce the incidence of TRAEs to a certain extent.

Similarly, the binding of the antibody to an appropriate target antigen is critically important. This requires that the antigen is preferentially or exclusively expressed on tumor cells, expressed at sufficient levels, exhibits high binding affinity, and is capable of internalizing the payload into the tumor cells. Therefore, identifying and selecting suitable antigens, as well as developing highly effective antibodies, can mechanistically reduce the occurrence of TRAEs. Concurrently, clinicians should familiarize themselves with the relevant immunohistochemical findings prior to treatment to make informed and rational selections regarding ADC therapy (Donaghy, 2016).

**FIGURE 5:**
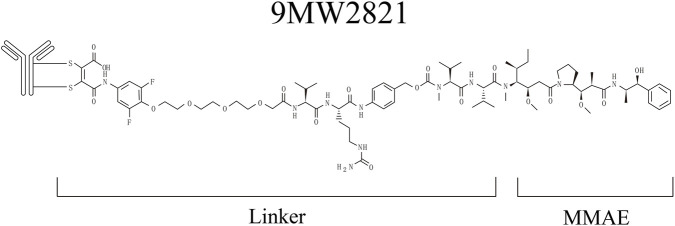
The molecular structural formula of 9MW2821. IDconnect utilizes novel interchain disulfide bond conjugation technology, resulting in a linker structure distinct from that of EV in Figure 2.

Finally, linker stability substantially impacts the systemic toxicity exerted by the payload. The development of more stable and specifically cleavable linkers remains a key pursuit in ADC research. A recent example is the novel Nectin-4-targeting ADC, 9MW2821/Bulumtatug fuvedotin (Figure 5). While structurally similar to EV, its distinguishing feature is the novel linker, IDconnect. Preclinical studies demonstrated that 9MW2821 exhibits higher *in vitro* serum stability and greater intratumoral MMAE exposure compared to EV (Fang et al., 2024; Zhou et al., 2023). Recent clinical trials of 9MW2821 have yielded preliminary results, showing promising efficacy and an acceptable safety profile across multiple solid tumors (Zhang J. et al., 2025). In addition to linkers, various components of novel ADCs are currently under development, including new payloads with simpler structures, lower molecular weights, and reduced toxicity and side effects, such as immune stimulants, PROTAC molecules, and photoactivated molecules. Moreover, bispecific ADCs, which offer greater precision and efficiency than traditional ADCs, and dual-drug ADCs with enhanced cytotoxic potency, are also being actively explored (Gu et al., 2024; Tsuchikama et al., 2024; Wang et al., 2023).

### 4.2 Clinical application of ADCs and management strategies for TRAEs

Second, from a clinical perspective, our findings indicate that the vast majority of TRAEs associated with ADCs targeting la/mUC can be managed through dose reduction or treatment discontinuation, with significant alleviation, improvement, or even complete resolution observed during subsequent follow-up. Therefore, active symptomatic management and well-timed prophylaxis before and during treatment are crucial. This review stratifies TRAEs according to the Common Terminology Criteria for Adverse Events (CTCAE) (DCTD, 2025), with Grade 5 events excluded from further discussion. Grade 1 events are generally considered manageable through monitoring, and, unless otherwise specified, will also not be discussed additionally. The common TRAEs for the three aforementioned ADCs and their corresponding management strategies are summarized in Supplementary Table S3. Certainly, for patients experiencing severe TRAEs, close monitoring of vital signs, along with guidance and intervention from relevant specialists, remains essential alongside active symptomatic treatment (Lacouture et al., 2022). For combination regimens involving ICIs, dose reduction or discontinuation of one or more agents should be implemented when necessary.

Despite the various TRAEs associated with ADCs used in la/mUC, a comprehensive review of current clinical trial results highlights several that warrant particular attention due to their association with dose reduction or treatment discontinuation, as well as a higher likelihood of being grade ≥3 TRAEs. Firstly, peripheral neuropathy, common with MMAE-based ADCs, is the most frequent reason for EV discontinuation. In severe cases, it can lead to muscle weakness, paralysis, visual changes, ptosis, dysphagia, myasthenia gravis, and Guillain-Barré syndrome (Brahmer et al., 2018). Moreover, it is a cumulative toxicity, typically emerging after the second treatment cycle and tending to worsen with continued therapy. Therefore, a comprehensive musculoskeletal assessment is recommended during clinical visits. Interventions such as physical therapy and the use of mechanical aids to address impaired balance and coordination may be considered. It is important to note that the management strategies outlined in the previous table generally have a slow onset of action, and patients should be informed that symptom relief may not be immediate (Pace et al., 2021).

Secondly, neutropenia represents the most common grade ≥3 TRAE and the leading cause of treatment discontinuation during SG use. In severe cases, it can manifest as febrile neutropenia and other infections secondary to neutropenia, potentially progressing to pancytopenia. Notably, the majority of severe neutropenia episodes occur within the first month of treatment (Powles et al., 2025). Spring et al. do not recommend the routine primary prophylaxis with G-CSF. However, they suggest secondary prophylaxis with G-CSF may be considered in cases of first occurrence of grade 4 neutropenia lasting ≥7 days, grade 3 febrile neutropenia, or grade 3–4 neutropenia requiring a dose delay of 2–3 weeks to recover to ≤ grade 1. They also recommend discontinuing treatment if such severe adverse events recur more than twice (Spring et al., 2021). In contrast, Tolaney et al. propose that primary G-CSF prophylaxis should also be administered to patients with poorer baseline conditions (e.g., age ≥65 years, prior neutropenia, poor performance status, organ dysfunction, or multiple comorbidities) (Tolaney et al., 2025). Currently, a consensus on the prophylactic use of G-CSF is still lacking (Pieniążek et al., 2025).

We have broadly summarized the causes of treatment-related deaths reported across different clinical trials. Due to variations in descriptions among researchers, the causes were generally categorized as follows: multiple organ dysfunction syndrome, septic shock, diabetic ketoacidosis, sepsis, pneumonia, pelvic abscess, febrile neutropenia, and neutropenic infection. Most of the deceased patients had poor baseline conditions or coexisting comorbidities. Furthermore, the majority of fatalities were associated with compromised anti-infective capacity and reduced immunity. Therefore, we believe that the prophylactic or targeted use of high-grade antibiotics may potentially reduce the incidence of treatment-related deaths. Additionally, close monitoring for potential TRAEs should commence early in the treatment course, as most cutaneous reactions and hyperglycemic events typically begin during the first treatment cycle (Yu et al., 2021).

Finally, as clinicians, it is essential to actively inquire about and monitor potential TRAEs during long-term follow-up of patients receiving therapy. ADC therapies are still in a relatively early stage of development, and their heterogeneous manifestations across different patient populations are not yet fully characterized, with some TRAEs potentially remaining unobserved or undocumented. Furthermore, with the increasing use of ADCs, the issue of drug resistance is becoming more prominent (Khoury et al., 2023). Clinicians should remain vigilant for signs of resistance during follow-up to avoid scenarios where patients continue to experience TRAEs without deriving clinical benefit due to developed resistance. Additionally, the mental health of patients during treatment should not be overlooked. Some patients may experience anxiety, panic, or even feelings of resignation due to the onset of TRAEs, while others might conceal or downplay symptoms for fear that their treatment might be interrupted or discontinued by their clinician. Fostering a positive patient understanding of TRAEs and encouraging open sharing of treatment experiences are crucial. This approach will facilitate a comprehensive understanding of disease progression and treatment response, ultimately supporting effective patient follow-up and management (Browe et al., 2024).

### 4.3 Emerging methods for TRAE risk stratification and prediction

We note that in recent years, studies have emerged enabling stratified prediction of TRAE risks. For example, Nectin-4 amplification has been identified as a genomic predictor of EV response and long-term survival in patients with mUC (Klümper et al., 2024). As mentioned earlier, patients homozygous for UGT1A1 allele variants have an increased risk of TRAEs such as neutropenia and diarrhea following SG treatment (Hulsh et al., 2022). In addition to the aforementioned biomarkers, pharmacokinetic/pharmacodynamic modeling has also become a research focus in recent years. It not only elucidates the exposure-response-toxicity relationship of ADCs *in vivo*, revealing the pharmacological underpinnings of interindividual heterogeneity in TRAEs, but also provides a rationale for implementing prospective dose individualization and optimizing dosing intervals to mitigate cumulative toxicity (Glassman, 2025). Furthermore, several novel real-time monitoring tools enable stratified prediction of TRAE risk through detection of targets of interest or clinical features, including circulating tumor DNA, soluble antigen levels, and patient-reported outcome platforms, among others (Hudgens et al., 2023; Morganti et al., 2025; Brouxhon et al., 2014).

## 5 Conclusion

EV, SG, and DV are main ADCs for the treatment of la/mUC, with their distinct targets and payloads determining their unique adverse event profiles. Although these TRAEs are largely manageable in clinical practice—with the majority of patients achieving symptom resolution or improvement through dose adjustments, symptomatic supportive care, and multidisciplinary collaboration—some severe events may still lead to treatment interruption or even life-threatening consequences. Clinicians should assess patients’ baseline status prior to treatment initiation, maintain close monitoring throughout therapy, and establish individualized intervention strategies. Meanwhile, the emergence of novel risk stratification tools offers new avenues for precision management of TRAEs, and optimized designs of next-generation ADCs hold promise for further reducing TRAEs incidence while maintaining therapeutic efficacy. In conclusion, comprehensive understanding of the TRAE characteristics, underlying mechanisms, and risk factors associated with different ADCs, mastery of standardized management strategies, and active application of emerging risk prediction tools are essential for achieving precision ADC therapy and maximizing patient benefit. In the future, as more clinical data accumulate and translational research deepens, ADC-based treatment for la/mUC will become increasingly personalized and safer.
