# Supplementary material for: Application of antibody-drug conjugates in locally advanced or metastatic urothelial carcinoma: mechanisms, treatment-related adverse events, and management strategies
Source: Front Pharmacol. 2026 Mar 31;17:1771653. doi: 10.3389/fphar.2026.1771653 (PMC13076500; doi:10.3389/fphar.2026.1771653)
Supplement: Supplementary file 1 [file Table1.docx]

**Table 1. Summary of Safety Outcomes from Relevant Clinical Studies**

| Clinical trial | Trial design | Common TRAEs | Grade ≥ 3 TRAEs | Discontinued & Dose reduced due to TRAEs |
| --- | --- | --- | --- | --- |
| EV Phase I, Japan (1) | EV | Dysgeusia and alopecia (52.9%), dry skin (47.1%) and pruritus (47.1%) | 10 (58.8%) | N/A |
| EV-101, Phase I (2) | EV | Fatigue (53%), alopecia (46%), and decreased appetite (42%) (n=112; EV: 1.25 mg/kg) | 53 (34%, n=155) | 16 (10%, n=155) &  3 (11%, n=27, EV: 1.00 mg/kg), 39 (35%, n=112; EV: 1.25 mg/kg) |
| EV-103，Phase I/II | Cohort A: EV+Pb  (3) | Peripheral sensory neuropathy (55.6%), fatigue (51.1%), and alopecia (48.9%) | 29 (64.4%) | 11 (24.5%) & 14 (31.1%) |
|  | Cohort K: EV vs EV+Pb (4) | EV+Pb only  Fatigue (56.6%), peripheral sensory neuropathy (51.3%), alopecia (46.1%) and rash maculopapular (46.1%) | 35 (47.9%) vs 48 (63.2%) | Discontinuation only  14 (19.2%) vs 19 (25.0%, EV only), 17 (22.4%, Pb only), 4 (5.3%, EV & Pb) |
| EV-201, Phase II | Cohort 1: after platinum and PD-1/PD-L1 inhibitors (5) | Fatigue (50%), alopecia (49%) and decreased appetite (44%) | 68 (54%) | 15 (12%) & 40 (32%) |
|  | Cohort 2: only after PD1/PDL1 inhibitors (6) | Alopecia (51%), peripheral sensory neuropathy (48%) and fatigue (34%) | 49 (55%) | 14 (16%) & 41 (46%) |
| EV-301, Phase III | EV vs chemotherapy (docetaxel, paclitaxel, or vinflunine) (Japan) (7) | Alopecia (52.8%), dysgeusia (50.0%) and peripheral sensory neuropathy (47.2%) vs neutropenia (50.0%), alopecia (37.5%) and WBC count decreased (37.5%) | 23 (63.9%) vs 36 (75.0%) | 9 (25%) vs 11 (22.9) & 14 (38.9%) vs 16 (33.3%) |
|  | EV vs chemotherapy (docetaxel, paclitaxel, or vinflunine) (8) | Alopecia (45.3%), peripheral sensory neuropathy (33.8%) and pruritus (32.1%) vs alopecia (36.4%), decreased appetite (23.4%) and fatigue (22.7%) | 152 (51.4%) vs 145 (49.8%) (52.4% vs 50.5% 24 mo) | 40 (13.5%) vs 33(11.3%) & 96 (32.4%) vs 80 (27.5%) |
| EV-302, Phase III (9) | EV & Pembrolizumab vs platinum-based chemotherapy | Peripheral sensory neuropathy (50.0%), pruritus (39.8%) and alopecia (33.2%) vs anemia (56.6%), neutropenia (41.6%) and nausea (38.8%) | 246 (55.9%) vs 301 (69.5%) | 154 (35.0%) vs 80 (18.5%) & 179 (40.7%) vs 164 (37.9%) |
| The double ADC, Phase I (10) | SG+EV | Diarrhea (87.0%), anemia (73.9%) and neutropenia (69.5%) | 18 (78%) | 2 (8.7%) & 11 (48%) |
| TROPHY-U-01, Phase II | Cohort 1: SG (11,12) | Diarrhea (65%), nausea (60%) and fatigue (52%) | 74 (65%) | 8 (7%) & 45 (40%) |
|  | Cohort 2: SG (13) | Diarrhea (66%), nausea (53%) and fatigue (50%) | 33 (87%) | 8 (21%) & 14(37%) |
|  | Cohort 3: SG & pembrolizumab (14) | Diarrhea (71%), nausea (56%) and neutropenia (51%) | 25 (61%) | 6 (15%) & 16 (39%) |
| TROPiCS-04, Phase III (15) | SG vs treatment of physician’s choice (TPC; paclitaxel, docetaxel, or vinflunine) | Fatigue (54%), diarrhea (52%) and neutropenia (48%) vs fatigue (39%), alopecia (33%) and anemia (29%) | 233 (67%) vs 119 (35%) | 39 (11%) vs 43 (12%) & 131 (37%) vs 93 (26%) |
| DV+PD-1 inhibitor, China (16–21) | DV+PD-1 inhibitor | Decreased appetite (33.3%), rash (33.3%) and weak (33.3%) | 2 (22.2%) | N/A |
|  | DV+PD-1 inhibitor | Hypaesthesia (31.3%), alopecia (25.0%) and leukopenia (25.0%) | 0 (0.00%) | N/A |
|  | DV+PD-1 inhibitor | Anorexia (78%), asthenia (55%) and rash (49%) | 2 (4%) | N/A |
|  | DV+PD-1 inhibitor | aspartate aminotransferase increased (65.9%), alanine aminotransferase increased (63.4%), and peripheral sensory neuropathy (63.4%) | 21 (51.2%) | 3 (7.3%) & 15 (36.6%) |
|  | DV+PD-1 inhibitor vs chemotherapy group (gemcitabine and cisplatin regimen) | Neutropenia (59.26%), thrombocytopenia (44.44%), and nausea (44.44%) vs neutropenia (92.31%), thrombocytopenia (61.54%) and nausea (80.77%) | 0 (0.00%) vs 19 (73.08%) | N/A |
|  | DV+PD-1 inhibitor | Peripheral sensory neuropathy (40.4%), fatigue (26.8%) and alopecia (25.3%) | 39 (19.7%) | 17 (8.6%) & 13 (6.6%) |
| DV, China (22,23) | DV | Hypoesthesia (60.5%), alopecia (55.8%) and leukopenia (55.8%) | 25 (58%) | Discontinuation only  11 (25.6%) |
|  | DV | Peripheral sensory neuropathy (68.2%), leukopenia (50.5%) and neutropenia (42.1%) | 58 (54.2%) | 1 (0.9%) & 32 (29.9%) |
| DV/DV+PD-1 inhibitor, China (24–29) | DV/DV+PD-1 inhibitor | Anemia (71.1%), anorexia (57.9%) and asthenia (52.6%) | 0 (0.00%) | Discontinuation only  5 (13.2%) |
|  | DV/DV+PD-1 inhibitor | Anemia (55.6%), hypoesthesia (33.3%) and fatigue (33.3%) | 6 (16.7%) | N/A |
|  | DV/DV+PD-1 inhibitor | Hypoesthesia (34.3%), anorexia (17.1%) and pruritus (14.3%) | 3 (7.1%) | Discontinuation only  3 (7.1%) |
|  | DV/DV+PD-1 inhibitor | Peripheral  sensory neuropathy (53.4%), alopecia (42.7%) and asthenia (38.8%) | 18 (17.5%) | Discontinuation only  16 (15.5%) |
|  | DV/DV+PD-1 inhibitor | Peripheral neuropathy (37.0%), anorexia (33.3%) and nausea (29.6%) | 4 (14.8%) | N/A |
|  | DV/DV+PD-1 inhibitor | Decreased appetite (54.5%), fatigue (36.4%) and cutaneous rash (36.4%) | 3 (27.3%) | N/A |

Reference

1. Takahashi S, Uemura M, Kimura T, Kawasaki Y, Takamoto A, Yamaguchi A, et al. A phase I study of enfortumab vedotin in Japanese patients with locally advanced or metastatic urothelial carcinoma. Invest New Drugs. 2020 Aug;38(4):1056–66. doi:10.1007/s10637-019-00844-x PubMed PMID: 31444589; PubMed Central PMCID: PMC7340645.

2. Rosenberg J, Sridhar SS, Zhang J, Smith D, Ruether D, Flaig TW, et al. EV-101: A Phase I Study of Single-Agent Enfortumab Vedotin in Patients With Nectin-4-Positive Solid Tumors, Including Metastatic Urothelial Carcinoma. J Clin Oncol Off J Am Soc Clin Oncol. 2020 Apr 1;38(10):1041–9. doi:10.1200/JCO.19.02044 PubMed PMID: 32031899; PubMed Central PMCID: PMC7106979.

3. Hoimes CJ, Flaig TW, Milowsky MI, Friedlander TW, Bilen MA, Gupta S, et al. Enfortumab Vedotin Plus Pembrolizumab in Previously Untreated Advanced Urothelial Cancer. J Clin Oncol. 2023 Jan 1;41(1):22–31. doi:10.1200/JCO.22.01643 PubMed PMID: 36041086; PubMed Central PMCID: PMC10476837.

4. O’Donnell PH, Milowsky MI, Petrylak DP, Hoimes CJ, Flaig TW, Mar N, et al. Enfortumab Vedotin With or Without Pembrolizumab in Cisplatin-Ineligible Patients With Previously Untreated Locally Advanced or Metastatic Urothelial Cancer. J Clin Oncol Off J Am Soc Clin Oncol. 2023 Sep 1;41(25):4107–17. doi:10.1200/JCO.22.02887 PubMed PMID: 37369081; PubMed Central PMCID: PMC10852367.

5. Rosenberg JE, O’Donnell PH, Balar AV, McGregor BA, Heath EI, Yu EY, et al. Pivotal Trial of Enfortumab Vedotin in Urothelial Carcinoma After Platinum and Anti-Programmed Death 1/Programmed Death Ligand 1 Therapy. J Clin Oncol. 2019 Oct 10;37(29):2592–600. doi:10.1200/JCO.19.01140 PubMed PMID: 31356140; PubMed Central PMCID: PMC6784850.

6. Yu EY, Petrylak DP, O’Donnell PH, Lee JL, van der Heijden MS, Loriot Y, et al. Enfortumab vedotin after PD-1 or PD-L1 inhibitors in cisplatin-ineligible patients with advanced urothelial carcinoma (EV‑201): a multicentre, single-arm, phase 2 trial. Lancet Oncol. 2021 Jun;22(6):872–82. doi:10.1016/S1470-2045(21)00094-2 PubMed PMID: 33991512.

7. Matsubara N, Yonese J, Kojima T, Azuma H, Matsumoto H, Powles T, et al. Japanese subgroup analysis of EV‐301: An open‐label, randomized phase 3 study to evaluate enfortumab vedotin versus chemotherapy in subjects with previously treated locally advanced or metastatic urothelial carcinoma. Cancer Med. 2022 Sep 2;12(3):2761–71. doi:10.1002/cam4.5165 PubMed PMID: 36052536; PubMed Central PMCID: PMC9939146.

8. Powles T, Rosenberg JE, Sonpavde GP, Loriot Y, Durán I, Lee JL, et al. Enfortumab Vedotin in Previously Treated Advanced Urothelial Carcinoma. N Engl J Med. 2021 Mar 25;384(12):1125–35. doi:10.1056/NEJMoa2035807 PubMed PMID: 33577729; PubMed Central PMCID: PMC8450892.

9. Powles T, Valderrama BP, Gupta S, Bedke J, Kikuchi E, Hoffman-Censits J, et al. Enfortumab Vedotin and Pembrolizumab in Untreated Advanced Urothelial Cancer. N Engl J Med. 2024 Mar 7;390(10):875–88. doi:10.1056/NEJMoa2312117 PubMed PMID: 38446675.

10. McGregor BA, Sonpavde GP, Kwak L, Regan MM, Gao X, Hvidsten H, et al. The Double Antibody Drug Conjugate (DAD) phase I trial: sacituzumab govitecan plus enfortumab vedotin for metastatic urothelial carcinoma☆. Ann Oncol. 2024 Jan 1;35(1):91–7. doi:10.1016/j.annonc.2023.09.3114 PubMed PMID: 37871703.

11. Loriot Y, Petrylak DP, Kalebasty AR, Fléchon A, Jain RK, Gupta S, et al. TROPHY-U-01, a phase II open-label study of sacituzumab govitecan in patients with metastatic urothelial carcinoma progressing after platinum-based chemotherapy and checkpoint inhibitors: updated safety and efficacy outcomes. Ann Oncol. 2024 Apr 1;35(4):392–401. doi:10.1016/j.annonc.2024.01.002 PubMed PMID: 38244927.

12. Tagawa ST, Balar AV, Petrylak DP, Kalebasty AR, Loriot Y, Fléchon A, et al. TROPHY-U-01: A Phase II Open-Label Study of Sacituzumab Govitecan in Patients With Metastatic Urothelial Carcinoma Progressing After Platinum-Based Chemotherapy and Checkpoint Inhibitors. J Clin Oncol. 2021 Aug 1;39(22):2474–85. doi:10.1200/JCO.20.03489 PubMed PMID: 33929895; PubMed Central PMCID: PMC8315301.

13. Petrylak DP, Tagawa ST, Jain RK, Bupathi M, Balar A, Kalebasty AR, et al. TROPHY-U-01 Cohort 2: A Phase II Study of Sacituzumab Govitecan in Cisplatin-Ineligible Patients With Metastatic Urothelial Cancer Progressing After Previous Checkpoint Inhibitor Therapy. J Clin Oncol. 2024 Oct 10;42(29):3410–20. doi:10.1200/JCO.23.01720 PubMed PMID: 39186707; PubMed Central PMCID: PMC11458109.

14. Grivas P, Pouessel D, Park CH, Barthelemy P, Bupathi M, Petrylak DP, et al. Sacituzumab Govitecan in Combination With Pembrolizumab for Patients With Metastatic Urothelial Cancer That Progressed After Platinum-Based Chemotherapy: TROPHY-U-01 Cohort 3. J Clin Oncol. 2024 Apr 20;42(12):1415–25. doi:10.1200/JCO.22.02835 PubMed PMID: 38261969; PubMed Central PMCID: PMC11095901.

15. Powles T, Tagawa S, Vulsteke C, Gross-Goupil M, Park SH, Necchi A, et al. Sacituzumab govitecan in advanced urothelial carcinoma: TROPiCS-04, a phase III randomized trial. Ann Oncol. 2025 May 1;36(5):561–71. doi:10.1016/j.annonc.2025.01.011 PubMed PMID: 39934055.

16. Ng C, Jing T, Yu S, Ye J, Zhang S, Jia Z, et al. The efficacy and safety of disitamab vedotin combined with immune checkpoint inhibitors in metastatic upper tract urothelial carcinoma: a multicenter real-world study. Cancer Immunol Immunother CII. 2025 Sep 13;74(10):304. doi:10.1007/s00262-025-04154-5 PubMed PMID: 40944727; PubMed Central PMCID: PMC12433416.

17. Wei Y, Zhang R, Yu C, Hong Z, Lin L, Li T, et al. Disitamab vedotin in combination with immune checkpoint inhibitors for locally and locally advanced bladder urothelial carcinoma: a two-center’s real-world study. Front Pharmacol. 2023 Aug 14;14:1230395. doi:10.3389/fphar.2023.1230395 PubMed PMID: 37645442; PubMed Central PMCID: PMC10461006.

18. Zhu K, Chang Y, Zhao D, Guo A, Cao J, Wu C, et al. Expression of HER2 in high-grade urothelial carcinoma based on Chinese expert consensus and the clinical effects of disitamab vedotin-tislelizumab combination therapy in the treatment of advanced patients. Front Pharmacol. 2024 Feb 22;15:1355081. doi:10.3389/fphar.2024.1355081 PubMed PMID: 38455962; PubMed Central PMCID: PMC10918465.

19. Yao JM, Zhong JL, Zhou Q, Guo J. Efficacy and safety of disitamab vedotin in combination with immune checkpoint inhibitors in patients with locally advanced or metastatic urothelial carcinoma. World J Urol. 2025 Mar 9;43(1):154. doi:10.1007/s00345-025-05544-1

20. Zhou L, Yang KW, Zhang S, Yan XQ, Li SM, Xu HY, et al. Disitamab vedotin plus toripalimab in patients with locally advanced or metastatic urothelial carcinoma (RC48-C014): a phase Ib/II dose-escalation and dose-expansion study. Ann Oncol. 2025 Mar 1;36(3):331–9. doi:10.1016/j.annonc.2024.12.002 PubMed PMID: 39662628.

21. Zhang T, He S, Tao L, Shi M, Wu Y, Guo Y, et al. Efficacy and safety of RC48 in combination with PD-1 inhibitors for the treatment of locally advanced or metastatic urothelial carcinoma: a single-center, real-world study. Discov Oncol. 2025 Apr 18;16:558. doi:10.1007/s12672-025-02362-0 PubMed PMID: 40249537; PubMed Central PMCID: PMC12008086.

22. Sheng X, Wang L, He Z, Shi Y, Luo H, Han W, et al. Efficacy and Safety of Disitamab Vedotin in Patients With Human Epidermal Growth Factor Receptor 2–Positive Locally Advanced or Metastatic Urothelial Carcinoma: A Combined Analysis of Two Phase II Clinical Trials. J Clin Oncol. 2024 Apr 20;42(12):1391–402. doi:10.1200/JCO.22.02912 PubMed PMID: 37988648; PubMed Central PMCID: PMC11095880.

23. Sheng X, Yan X, Wang L, Shi Y, Yao X, Luo H, et al. Open-label, Multicenter, Phase II Study of RC48-ADC, a HER2-Targeting Antibody–Drug Conjugate, in Patients with Locally Advanced or Metastatic Urothelial Carcinoma. Clin Cancer Res. 2021 Jan 1;27(1):43–51. doi:10.1158/1078-0432.CCR-20-2488

24. Wang A, Chen M, Li D, Shi J, Tang W, Zhang Z, et al. Disitamab Vedotin Alone or in Combination With Immune Checkpoint Inhibitors in Bladder-Sparing Treatment of Muscle-Invasive Bladder Cancer: A Real-World Study. Clin Genitourin Cancer. 2024 Jun;22(3):102085. doi:10.1016/j.clgc.2024.102085 PubMed PMID: 38636170.

25. Xu J, Zhang H, Zhang L, Chu X, Li Y, Li G, et al. Real‐world effectiveness and safety of RC48‐ADC alone or in combination with PD‐1 inhibitors for patients with locally advanced or metastatic urothelial carcinoma: A multicenter, retrospective clinical study. Cancer Med. 2023 Nov 7;12(23):21159–71. doi:10.1002/cam4.6680 PubMed PMID: 37935113; PubMed Central PMCID: PMC10726858.

26. Chen M, Yao K, Cao M, Liu H, Xue C, Qin T, et al. HER2-targeting antibody–drug conjugate RC48 alone or in combination with immunotherapy for locally advanced or metastatic urothelial carcinoma: a multicenter, real-world study. Cancer Immunol Immunother CII. 2023 Mar 10;72(7):2309–18. doi:10.1007/s00262-023-03419-1 PubMed PMID: 36897337; PubMed Central PMCID: PMC10264489.

27. Ge H, Liu C, Shen C, Hu D, Zhao X, Wang Y, et al. The effectiveness and safety of RC48 alone or in combination with PD-1 inhibitors for locally advanced or metastatic urothelial carcinoma: a multicenter, real-world study. J Transl Med. 2025 Feb 28;23:243. doi:10.1186/s12967-025-06237-4 PubMed PMID: 40022107; PubMed Central PMCID: PMC11871675.

28. Chen J, Wang M, Qi X, Long H, Qi N, Wu L, et al. RC48-Antibody-Drug Conjugate in Metastatic Urothelial Carcinoma: A Multicenter Real-World Study in China. Clin Genitourin Cancer. 2024 Jun;22(3):102093. doi:10.1016/j.clgc.2024.102093 PubMed PMID: 38762350.

29. Wang D, Cao M, Zhang Y, Bi L, Chen M, Ni M, et al. RC48-ADC monotherapy or in combination with immunotherapy for locally advanced or metastatic urothelial carcinoma with HER2 low and null expression: a multicenter, real-world, retrospective study. BMC Cancer. 2025 Apr 30;25:812. doi:10.1186/s12885-025-14154-4 PubMed PMID: 40307755; PubMed Central PMCID: PMC12044827.
