# Supplementary material for: Application of antibody-drug conjugates in locally advanced or metastatic urothelial carcinoma: mechanisms, treatment-related adverse events, and management strategies
Source: Front Pharmacol. 2026 Mar 31;17:1771653. doi: 10.3389/fphar.2026.1771653 (PMC13076500; doi:10.3389/fphar.2026.1771653)
Supplement: Supplementary file 3 [file Table3.docx]

Table 3 Summary of Management Strategies for TRAEs

| Common TRAEs | CTCAE grade | Management strategies |
| --- | --- | --- |
| Peripheral sensory neuropathy / hypaesthesia | 2 (1,2) | Gabapentin and pregabalin; duloxetine and venlafaxine; amitryptyline and nortriptyline; transient discontinue treatment until grade ≤1, resume treatment at the same dose level for the 1^st^ occurrence or reduced dose level for recurrent grade 2 |
|  | 3 & 4 (1) | Permanently discontinue treatment; continuation of the above symptomatic treatment |
| Dermatologic events, exemplified by rash (1,3) | 1 or asymptomatic | Prophylactic use of barrier-protecting agents such as zinc-containing moisturizers; ultraviolet radiation protection with sunscreen; topical steroids, antihistamines and antibiotics |
|  | 2 | Topical steroids, antihistamines, emollients and antibiotics; consider anti-infectives if necessary |
|  | 3 | Systemic steroids; transient discontinue treatment until grade ≤1, then resume treatment at the same dose level or dose reduce by one dose level; continuation of the above symptomatic treatment |
|  | 4 & recurrent grade 3 & confirmed stevens-Johnson syndrome and toxic epidermal necrolysis (SJS & TEN) | Permanently discontinue treatment; continuation of the above symptomatic treatment  (suspected SJS & TEN: immediately discontinue treatment for further diagnostic evaluation) |
| Neutropenia (4) | 1 or asymptomatic | Primary prophylactic use of G-CSF (0.5 MU/kg/day subcutaneously on cycle days 3, 4, 10, and 11) |
|  | 2 | G-CSF (5 μg/kg/day) |
|  | (Grade 4 neutropenia ≥ 7 days) OR (Grade 3–4 febrile neutropenia) OR (At time of scheduled dose, grade 3–4 neutropenia that delays dosing by 2–3 weeks for recovery to grade ≤ 1) | 1^st^ occurrence: dose reduction by 25% and administer G-CSF |
|  |  | 2^nd^ occurrence: dose reduction by 50% and administer G-CSF |
|  |  | 3^rd^ occurrence: discontinue treatment and administer G-CSF |
|  | At the time of scheduled dose, grade 3–4 neutropenia that delays SG dosing beyond 3 weeks for recovery to grade ≤ 1 | 1^st^ occurrence: discontinue treatment and administer G-CSF |
|  | Postscript | The initiation of G-CSF therapy required an assessment of complete blood count prior to the therapy’s commencement and through twice-weekly monitoring during the treatment course |
| Gastrointestinal events, exemplified by diarrhea, nausea and vomiting (4) | 1 or asymptomatic | Primary prophylactic loperamide for the management of diarrhea (2 mg twice a day or 4 mg once a day orally on cycle days 2, 3, 4, 9, 10, and 11 during at least the first two cycles); evaluate for infectious causes |
|  | 2 | Loperamide for the management of diarrhea; dexamethasone, a 5-hydroxytryptamine receptor antagonist (5-HT3 RA), and a neurokinin 1 receptor antagonist (NK-1 RA), and olanzapine if necessary for nausea and vomiting |
|  | Diarrhea (grade 3 or 4) | Loperamide: 4 mg initially, followed by 2 mg with every episode of diarrhea to a maximum of 16 mg daily; discontinue loperamide 12 h after diarrhea resolves; discontinue treatment until diarrhea is resolved to grade ≤ 1 |
|  | Nausea and vomiting (grade 3 or 4) | Premedication: A 2–3 drug combination regimen 30 to 60 min prior to ADC infusion (e.g., dexamethasone with either a 5-HT3 RA or an NK-1 RA, as well as other drugs, as indicated); dexamethasone 8 mg or 12 mg po; ondansetron 8 mg po 30 to 60 mg minutes prior to ADC; aprepitant 125 mg po and ondansetron 8 mg IV or po; netupitant/palonosetron 300 mg–0.5 mg po |
|  | (Grade 4 diarrhea, nausea, or vomiting) OR (Grade 3 diarrhea, nausea, or vomiting not controlled by medication or persisting for >48 h despite optimal medical management) OR (At time of scheduled treatment, grade 3–4 diarrhea, nausea, or vomiting which delays dose by 2–3 weeks for recovery to grade ≤ 1) | 1^st^ occurrence: dose reduction by 25%; continuation of the above symptomatic treatment |
|  |  | 2^nd^ occurrence: dose reduction by 50%; continuation of the above symptomatic treatment |
|  |  | 3^rd^ occurrence: discontinue treatment; continuation of the above symptomatic treatment |
|  | Grade 3–4 diarrhea, nausea, or vomiting which does not recover to grade ≤ 1 within 3 weeks | 1^st^ occurrence: discontinue treatment; continuation of the above symptomatic treatment |
|  | Postscript | Attention for supportive measures: Fluid and electrolyte replacement; traditional Chinese medicine may be considered as a treatment option (5) |
| Hyperglycemia (1) | 1 | Insulin therapy; anti-hyperglycemic as clinically indicated |
|  | 2 & 3 & 4 | Transient discontinue treatment until elevated blood glucose has improved to ≤250 mg/dL, (13.9mmol/L); resume treatment at the same dose level; continuation of the above symptomatic treatment |
|  | Postscript | Blood glucose>500mg/dL: immediately discontinue treatment for further diagnostic evaluation; attention for diabetic ketoacidosis (DKA) |
| Hepatotoxicity (6) | 2 | Consider dose reduction; glycyrrhizic acid preparations and ursodeoxycholic acid tablets |
|  | 3 & 4  (ALT or AST >8 × upper limit) OR (ALT or AST >5 × upper limit, lasting for 2 weeks) OR (ALT or AST >3 × upper limit with TBil >2 × upper limit or International Normalized Ratio >1.5) OR (ALT or AST >3 × upper limit with a gradual worsening of fatigue and gastrointestinal symptoms) | Immediately discontinue treatment or dose reduction if necessitated by treatment of the primary disease and the lack of other alternative options.; continuation of the above symptomatic treatment |
| Pneumonitis (1) | 2 | Immediately discontinue treatment until grade ≤1; administer steroids; resume treatment at the same dose level or consider dose reduction by one dose level |
|  | 3 & 4 | Permanently discontinue treatment; continuation of the above symptomatic treatment; consider hospitalization |
| Anemia (7) | 2 | Transfusion of packed red blood cells for those with hemoglobin below 7 g/dL (70g/L); erythropoiesis-  colony stimulating agents (use with caution) |
|  | 3 & 4 | Dose reduction or treatment discontinuation for those with severe or persistent anemia (＜60 g/L; ＞2~4 weeks); continuation of the above symptomatic treatment |

Reference

1. Brower B, McCoy A, Ahmad H, Eitman C, Bowman IA, Rembisz J, et al. Managing potential adverse events during treatment with enfortumab vedotin + pembrolizumab in patients with advanced urothelial cancer. Front Oncol. 2024 Apr 22;14:1326715. doi:10.3389/fonc.2024.1326715 PubMed PMID: 38711854; PubMed Central PMCID: PMC11071165.

2. Mauermann ML, Staff NP. Peripheral Neuropathy: A Review. JAMA. 2026 Jan 20;335(3):255–66. doi:10.1001/jama.2025.19400 PubMed PMID: 41247746.

3. Lacouture ME, Patel AB, Rosenberg JE, O’Donnell PH. Management of Dermatologic Events Associated With the Nectin-4-directed Antibody-Drug Conjugate Enfortumab Vedotin. The Oncologist. 2022 Mar 11;27(3):e223–32. doi:10.1093/oncolo/oyac001 PubMed PMID: 35274723; PubMed Central PMCID: PMC8914492.

4. Manna M, Brabant M, Greene R, Chamberlain MD, Kumar A, Alimohamed N, et al. Canadian Expert Recommendations on Safety Overview and Toxicity Management Strategies for Sacituzumab Govitecan Based on Use in Metastatic Triple-Negative Breast Cancer. Curr Oncol. 2024 Sep 21;31(9):5694–708. doi:10.3390/curroncol31090422 PubMed PMID: 39330050; PubMed Central PMCID: PMC11431578.

5. Lin X, Fang Y, Cheng Y, Wang Q. Chinese herbal medicine for irinotecan-induced diarrhea: A systematic review and meta-analysis. Explore. 2024;20(2):158–67. doi:10.1016/j.explore.2023.08.003 PubMed PMID: 37640591.

6. Yang P, Zhang H, Li J, Li Z, Liu Z, Wang M, et al. Incidence of antibody–drug conjugate-related hepatotoxicity in breast cancer: a systematic review and meta-analysis. Ther Adv Drug Saf. 2024 Dec 18;15:20420986241304680. doi:10.1177/20420986241304680 PubMed PMID: 39703774; PubMed Central PMCID: PMC11656431.

7. Schlam I, Tarantino P, Tolaney SM. Managing adverse events of sacituzumab govitecan. Expert Opin Biol Ther. 2023;23(11):1103–11. doi:10.1080/14712598.2023.2267975 PubMed PMID: 37800595.
